# The diagnostic accuracy of urine-based tests for bladder cancer varies greatly by patient

**DOI:** 10.1186/s12894-016-0147-5

**Published:** 2016-06-13

**Authors:** Ajay Gopalakrishna, Thomas A. Longo, Joseph J. Fantony, Richmond Owusu, Wen-Chi Foo, Rajesh Dash, Brant A. Inman

**Affiliations:** Division of Urology, Duke University Medical Center, Durham, NC 27710 USA; Department of Urology, University of California San Diego, San Diego, CA USA; Department of Pathology, Duke University Medical Center, Durham, NC USA

**Keywords:** FISH, Cytology, Bladder cancer, Sensitivity, Specificity, Spectrum effects

## Abstract

**Background:**

Spectrum effects refer to the phenomenon that test performance varies across subgroups of a population. When spectrum effects occur during diagnostic testing for cancer, difficult patient misdiagnoses can occur. Our objective was to evaluate the effect of test indication, age, gender, race, and smoking status on the performance characteristics of two commonly used diagnostic tests for bladder cancer, urine cytology and fluorescence in situ hybridization (FISH).

**Methods:**

We assessed all subjects who underwent cystoscopy, cytology, and FISH at our institution from 2003 to 2012. The standard diagnostic test performance metrics were calculated using marginal models to account for clustered/repeated measures within subjects. We calculated test performance for the overall cohort by test indication as well as by key patient variables: age, gender, race, and smoking status.

**Results:**

A total of 4023 cystoscopy-cytology pairs and 1696 FISH-cystoscopy pairs were included in the analysis. In both FISH and cytology, increasing age, male gender, and history of smoking were associated with increased sensitivity and decreased specificity. FISH performance was most impacted by age, with an increase in sensitivity from 17 % at age 40 to 49 % at age 80. The same was true of cytology, with an increase in sensitivity from 50 % at age 40 to 67 % at age 80. Sensitivity of FISH was higher for a previous diagnosis of bladder cancer (46 %) than for hematuria (26 %). Test indication had no impact on the performance of cytology and race had no significant impact on the performance of either test.

**Conclusions:**

The diagnostic performance of urine cytology and FISH vary significantly according to the patient demographic in which they were tested. Hence, the reporting of spectrum effects in diagnostic tests should become part of standard practice. Patient-related factors must contextualize the clinicians’ interpretation of test results and their decision-making.

## Background

Bladder cancer (BC) represents 4.5 % of all new cancers in the US with over 74,000 cases and it remains the 5th most common in 2015 [1]. Typically, it presents with hematuria, and 70 % of patients with BC initially have non-muscle invasive bladder cancer (NMIBC). NMIBC has a high chance of recurrence (60–85 %) and requires long term surveillance [2]. Several guidelines exist for the management of non-muscle invasive bladder cancer, and include cystoscopy and urine-based tests for initial screening and recurrence surveillance [3–5].

Cystoscopy is the community gold standard for the detection of bladder tumors, and identifies nearly all papillary and sessile tumors [6]. However, it is invasive and a source of distress for patients. It also has a limited ability to detect occult microscopic disease or the presence of tumors in atypical locations. Microscopic disease is of particular importance in BC because of prevalent field effect [7]. While urethral cancer is a rare event, [8] upper tract tumors (UTUC) account for 5–10 % of urothelial cell carcinoma and may lead to increased morbidity and mortality if missed [9]. Therefore, guidelines recommend adjunctive tests for detection of BC [3–5]. The two most common urine-based tests are voided urine cytology and UroVysion™ (Vysis, Downers Grove, IL) fluorescence in situ hybridization (FISH) assay. Most physicians and their patients will assume that a positive urine test indicates the presence of a tumor, and will aggressively pursue a diagnosis.

The majority of physicians believe that a urine test will perform similarly in all patient populations, but this may be a false assumption. Test performance often varies across patient subgroups and is termed spectrum effects [10–12]. Although reporting spectrum effects for a given test is endorsed by the STARD initiative, it is uncommon in practice [13]. We are the first to evaluate for the existence of spectrum effects in cytology and FISH among patients being screened because of hematuria or undergoing surveillance of NMIBC. Our hypothesis is that test performance varies according to patient characteristics. We analyzed the diagnostic performance by test indication as well as four clinically significant demographic variables - age, gender, race, and smoking status. The objective of this study was to determine the presence and magnitude of spectrum effects occurring in cytology and FISH of a large contemporary cohort undergoing bladder cancer screening.

## Methods

### Subject selection

After approval by the Duke University Health System Institutional Review Board, all subjects who underwent cystoscopy and cytology and/or UroVysion FISH at Duke University Medical Center (DUMC) between 1/2003 to 1/2012 for either hematuria evaluation or surveillance of bladder cancer were identified. As the data for the study was obtained through retrospective chart review, a waiver of informed consent was approved by the IRB. For patients with signs or symptoms of urinary tract infection, the standard practice at our institution was to collect a urine specimen for culture, treat the patient with culture-specific antibiotics, and delay cystoscopy and urine marker testing for 2–4 weeks to avoid confounding the results.

### Cystoscopy as the diagnostic gold standard for bladder tumor

White light cystoscopy, the community gold standard in diagnosis of bladder tumors, was used to determine the presence or absence of a bladder tumor [4, 14–16]. Cystoscopy was chosen over biopsy as the standard against which urine tests were compared because a biopsy is obtained only in subjects with an abnormal cystoscopy or urine test, which would subject the results to considerable verification bias [17]. Cystoscopy results were classified as positive, suspicious, or negative. A positive cystoscopy serves as a surrogate for histopathology, as nearly all visible tumors are malignant [6]. We required that cystoscopy occur within +/− 30 days of the urine-based test to serve as the gold standard.

### Cytology

Urine samples received in the Cytology Preparatory Laboratory were prepared as ThinPrep slides (Cytyc Corporation, Marlborough, MA). After samples were centrifuged at 2800 rpm for 5 min, the supernatant was removed to produce a cell pellet. Cell pellets were washed with Cytolyt Solution. Two to three drops of each patient sample was transferred into PreservCyt Solution and fixed for 15 min. ThinPrep slides were then produced by loading the samples into the ThinPrep 2000 Processor. The ThinPrep slides were stained with Papanicolaou stain, cover-slipped and then screened by a cytotechnologist before being evaluated by a cytopathologist. More than one cytopathologist was involved in the analysis of the urine specimens during the study interval. After cytological evaluation, the specimens were classified into one of four categories: negative, atypical, suspicious for malignancy, or positive for malignancy.

### UroVysion FISH test

Patient samples for UroVysion FISH were prepared according to manufacturer recommendations (Abbott Molecular Inc., Abbot Park, IL). The UroVysion Probe mixture contains chromosome enumeration probes (CEPs) labeled with Spectrum Red for visualization of chromosome 3, Spectrum Green for visualization of chromosome 7 and Spectrum Aqua for visualization of chromosome 17, as well as a locus specific probe for 9p21 labeled with Spectrum Gold. The slides were counterstained with DAPI and visualized with a fluorescence microscope equipped with the appropriate filters for signal enumeration of each fluorophore. A minimum of 25 morphologically abnormal cells per test were analyzed. The UroVysion FISH result was defined as meeting one or more of the following criteria: (i) ≥ 4 cells with gains of 2 or more chromosomes 3, 7, and 17 in the same cell, (ii) ≥ 10 cells with tetrasomy of chromosomes 3, 7, and 17, (iii) ≥ 10 cells showing gains of a single chromosome 3, 7, or 17, and (iv) ≥ 12 cells with homozygous loss of 9p21 locus [18].

### Statistical methods

Diagnostic test performance metrics and 95 % confidence intervals (95 % CI) were calculated using logistic models: (a) a generalized estimating equation (GEE) using an exchangeable (compound symmetry) covariance structure, [19] and (b) a generalized linear mixed model (GLMM) [19]. While both models take into account clustered/correlated test results that occur due to repeated testing within subjects, they are different techniques and results are interpreted differently [20]. The GEE is a marginal model that is interpreted as “population-averaged,” whereas the GLMM is a conditional model interpreted in a “subject-specific” manner [21]. Sensitivity and specificity were calculated for the overall cohort as well as by indication, age, gender, race, and smoking status subgroups. Age was analyzed as a continuous variable, but the results are presented in age decades for ease of interpretation. Indication, gender, race, and smoking status were analyzed as categorical variables. Smokers were stratified as “Never smokers,” “Former smokers,” or “Current smokers,” as indicated in their electronic medical charts. Smoking status was available on all patients in both the cytology and FISH cohorts. A two-sided *p*-value of 0.05 was used to define statistical significance. Statistical analyses were conducted using R 3.1.3 with packages lme4, geepack, and BSagri installed.

## Results

A total of 4023 pairs of cystoscopies and cytologies were obtained from 871 unique subjects for the cytology analysis, and 1696 pairs of UroVysion tests and cystoscopies from 827 unique subjects for the UroVysion FISH analysis. Baseline demographic characteristics of the study cohort are shown in Table 1. In patients who had positive pathology in the cytology cohort, the AJCC stage distribution was: 355 (81 %) stage 0, 33 (7.5 %) stage 1, 33 (7.5 %) stage 2, and 19 (4 %) stage 3. The grade distribution was 199 (45 %) low grade and 239 (55 %) high grade. In the FISH cohort, of patients who had positive pathology, the AJCC stage distribution was: 183 (77 %) stage 0, 24 (10 %) stage 1, 18 (8 %) stage 2, 12 (5 %) stage 3, and 1 (<1 %) stage 4. The grade breakdown was 102 (43 %) low grade and 134 (56 %) high grade.

**Table 1:** Clinical characteristics of the study population

	Cytology cohort	UroVysion FISH cohort
Sample size		
Unique subjects	871	827
Test-cystoscopy pairs	4023	1696
Age (years, median)	66 (IQR: 56–75)	67 (IQR: 56–76)
< 40	34 (4 %)	29 (4 %)
40–50	89 (10 %)	84 (10 %)
50–60	162 (19 %)	152 (18 %)
60–70	248 (29 %)	227 (27 %)
70–80	222 (26 %)	217 (26 %)
≥ 80	113 (13 %)	118 (14 %)
Gender		
Male	540 (62 %)	488 (59 %)
Female	328 (38 %)	339 (41 %)
Race		
White	668 (78 %)	648 (78 %)
Black	157 (18 %)	154 (19 %)
Other	26 (3 %)	25 (3 %)
Smoking status		
Current smoker	75 (9 %)	82 (10 %)
Former smoker	403 (48 %)	407 (49 %)
Never smoker	356 (43 %)	338 (41 %)
Indication for test		
Hematuria	415 (48 %)	368 (44 %)
Urothelial carcinoma	331 (38 %)	322 (39 %)
Other	125 (14 %)	137 (17 %)
Cystoscopy result		
Negative	2783 (69 %)	1324 (78 %)
Positive	752 (19 %)	185 (11 %)
Atypical/Suspicious	492 (12 %)	187 (11 %)
Urine test result		
Negative	1632 (41 %)	1210 (71 %)
Positive	375 (9 %)	486 (29 %)
Atypical/Suspicious	2016 (50 %)	-

*IQR* interquartile range

**Fig. 1:**
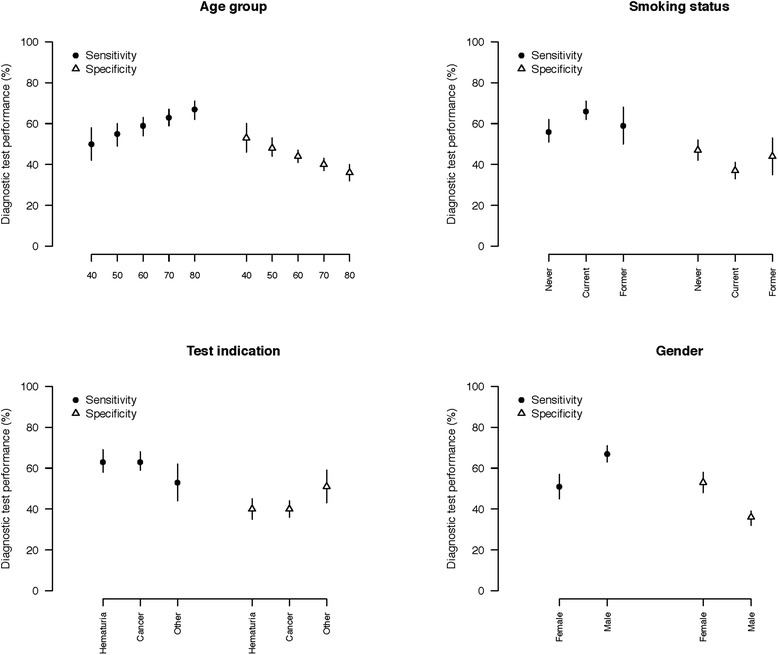
Cytology test performance characteristics by patient subgroups

The diagnostic performance of urine cytology is shown in Table 2 and Fig. 1. Increasing age was associated with an increase in sensitivity and decrease in specificity of urine cytology. Sensitivity increased by 17 %, from 50 % in subjects ≤40 years to 67 % in those ≥80 years. In contrast, specificity declined from 53 % in subjects ≤40 years of age to 36 % in subjects ≥80 years of age. Gender had the greatest impact on cytology performance. Subject-specific estimates of sensitivity derived from the GLMM model were dramatically higher in men than women (67 % vs 51 %), though specificity was lower (36 % vs 53 %). In subjects with a history of smoking, cytology was 10 % more sensitive and proportionally less specific compared with subjects who had never smoked. Race and indication did not significantly impact cytology test performance in either of the models.

**Table 2:** Diagnostic performance of urine cytology by patient subgroup

Risk factor	Subgroup	Method	Sensitivity			Specificity			*P*-value
			Estimate (%)	LCI	UCI	Estimate (%)	LCI	UCI	
Overall	None	GLMM	62	58	66	41	38	44	0.13
		GEE	59	56	63	43	40	45	0.23
Age	40	GLMM	50	42	58	53	46	60	<0.001
	50		55	49	60	48	44	53	
	60		59	54	63	44	41	47	
	70		63	59	67	40	37	43	
	80		67	62	71	36	32	40	
	40	GEE	50	43	57	52	46	58	0.006
	50		53	48	59	49	45	53	
	60		57	53	61	45	42	48	
	70		60	57	64	42	39	44	
	80		64	60	68	38	35	42	
Smoking	Never	GLMM	56	51	62	47	42	52	0.003
	Former		66	62	71	37	33	41	
	Current		59	50	68	44	35	53	
	Never	GEE	55	50	60	47	43	51	0.005
	Former		63	59	67	39	36	43	
	Current		57	49	65	45	38	52	
Gender	Female	GLMM	51	45	57	53	48	58	<0.001
	Male		67	63	71	36	32	39	
	Female	GEE	50	45	55	52	48	56	<0.001
	Male		64	60	67	38	35	41	
Race	White	GLMM	63	59	67	40	37	44	0.34
	Black		61	53	68	43	36	50	
	Other		51	34	68	52	36	68	
	White	GEE	60	56	64	42	39	45	0.25
	Black		58	51	65	44	38	50	
	Other		49	35	63	53	39	67	
Indication	Hematuria	GLMM	63	58	69	40	35	45	0.047
	Cancer		63	59	68	40	36	44	
	Other		53	44	62	51	43	59	
	Hematuria	GEE	61	56	65	42	38	46	0.059
	Cancer		60	56	64	42	39	45	
	Other		52	45	59	51	44	57

**Fig. 2:**
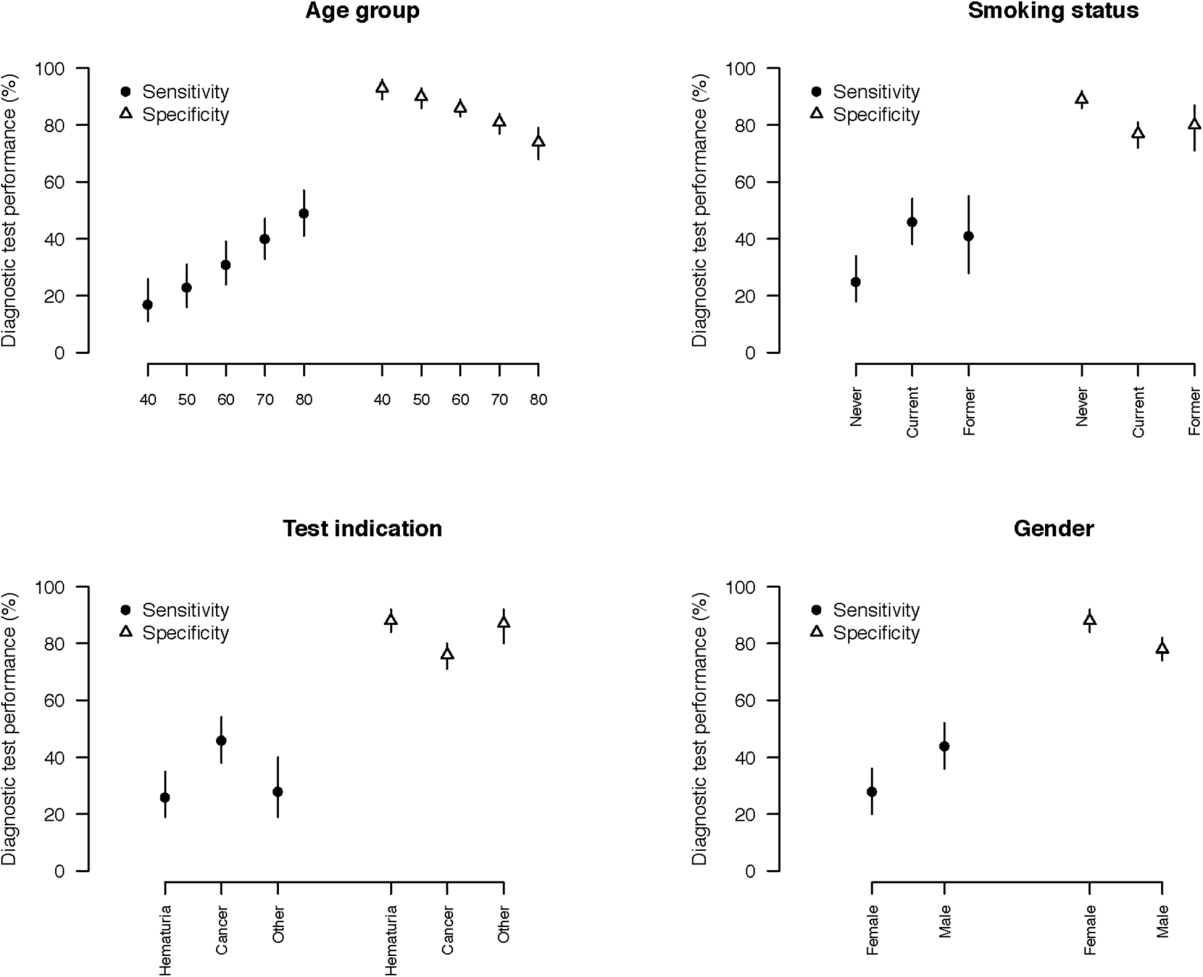
UroVysion FISH test performance characteristics by patient subgroups

The diagnostic performance of UroVysion FISH is shown in Table 3 and Fig. 2. Again, increasing subject age was associated with increased sensitivity and decreased specificity. Subject-specific estimates of test sensitivity obtained from the GLMM model nearly tripled from 17 % in subjects ≤40 years of age to 49 % in those ≥80 years of age. Contrarily, specificity decreased from 93 % in subjects ≤40 years of age to 74 % in those ≥80 years of age. The UroVysion FISH test was substantially less sensitive in women than in men (28 % vs. 44 %), though its specificity was higher (88 % vs 78 %). Test performance was similar in current and former smokers regardless of the analysis model. However, in nonsmokers, test sensitivity was approximately 15 % lower and specificity approximately 10 % higher than current and former smokers. Race was not statistically significant in the correlative models. Analysis of test performance by indication revealed significant differences. FISH was dramatically more sensitive for cancer surveillance (46 %) than for hematuria (26 %). However, it was also less specific (76 % vs 88 %).

**Table 3:** Diagnostic performance of UroVysion FISH by patient subgroup

Risk factor	Subgroup	Method	Sensitivity			Specificity			*P*-value
			Estimate (%)	LCI	UCI	Estimate (%)	LCI	UCI	
Overall	None	GLMM	39	31	46	82	79	85	<0.001
		GEE	38	32	45	77	75	80	<0.001
Age	40	GLMM	17	11	26	93	89	96	<0.001
	50		23	16	31	90	86	93	
	60		31	24	39	86	83	89	
	70		40	33	47	81	77	84	
	80		49	41	57	74	68	79	
	40	GEE	20	14	28	89	85	92	<0.001
	50		25	19	33	86	82	89	
	60		32	26	39	81	78	84	
	70		39	33	46	76	73	79	
	80		47	40	54	70	65	74	
Smoking	Never	GLMM	25	18	34	89	86	92	<0.001
	Former		46	38	54	77	72	81	
	Current		41	28	55	80	71	87	
	Never	GEE	26	34	85	85	81	88	<0.001
	Former		44	52	72	72	68	75	
	Current		40	51	76	76	68	82	
Gender	Female	GLMM	28	20	36	88	84	92	<0.001
	Male		44	36	52	78	74	82	
	Female	GEE	28	22	36	84	80	87	<0.001
	Male		43	37	37	73	69	77	
Race	White	GLMM	40	33	47	81	77	85	0.219
	Black		31	21	42	87	81	91	
	Other		37	17	61	83	65	93	
	White	GEE	39	33	46	76	73	79	0.160
	Black		31	23	41	82	76	87	
	Other		35	17	58	80	61	91	
Indication	Hematuria	GLMM	26	19	35	88	84	92	<0.001
	Cancer		46	38	54	76	71	80	
	Other		28	19	40	87	80	92	
	Hematuria	GEE	29	22	37	83	79	86	<0.001
	Cancer		44	38	52	71	67	75	
	Other		31	22	41	82	75	87

There were 4,729 total cytologies collected, although 706 did not have a corresponding cystoscopy to perform the above analysis. During the study period, 1898 (40 %) were negative, 423 (9 %) positive, and 2408 (51 %) suspicious or atypical. When suspicious/atypical cytology results using the GLMM model were classified as positive, the sensitivity was 62 % [95 % CI: 58–66 %] and the specificity was 41 % [95 % CI: 38–44 %]. When these results were re-classified as negative, this had the effect of a large increase in specificity 100 % [95 % CI: 100-100 %] with a consequent decrease in sensitivity 0 % [95 % CI: 0-2 %].

For all the above analyses, suspicious cystoscopies were considered positive since they will generally result in intervention (e.g., bladder biopsy). To determine whether the classification of suspicious cystoscopies dramatically affected our results, we repeated the analyses with suspicious cystoscopies classified as negative and found no significant difference in our results, demonstrating that the performance of cytology and UroVysion FISH are not sensitive to how suspicious cystoscopies are classified. This stands in contrast to the large effect seen in cytology with a similar re-analysis that was mentioned above.

## Discussion

Spectrum effects were first described by Mulherin et al. as inherent variations in diagnostic test performance among different subgroup populations [12]. We have shown that urine-based tests for bladder cancer (a) have poor diagnostic performance and (b) vary substantially in accuracy in different patient populations. However, the recognition of spectrum effects allows for a strategy that should result in a clinically important gain for the patient.

We stratified our cohort into four clinically relevant subgroups and found that age, male gender, and a history of smoking were all associated with increased sensitivity in both cytology and UroVysion. Smoking and aging are associated with altered cellular biology which might lead to changes detectable by cytology or UroVysion [22]. Epidemiologically, age and cigarette smoking have also been associated with more advanced disease at initial presentation [22, 23]. It is possible that the improvement in sensitivity of cytology and UroVysion is due to more advanced disease at presentation in these demographics. Horstmann et al. found that age was associated with higher false positive rates in cytology and the NMP22 assay, which would translate to decreased specificity and is consistent with our results [24]. The analysis by indication also revealed increased sensitivity for UroVysion but not cytology when used for cancer surveillance compared to hematuria. This may also be a reflection of advanced disease in that population. Interestingly, Dimashkieh et al. found that both UroVysion and cytology are slightly more sensitive in the context of cancer surveillance than in hematuria [25].

Disease severity fails to explain why both tests were more sensitive in males than females. While the incidence of bladder cancer is three to four times higher in men, women tend to present with more advanced disease [26, 27]. An alternative explanation for the gender disparity we observed is that gender-specific genetic differences are affecting test performance. Recent studies have found gender differences at a cellular level, and postulate that cells have a “sex” [28]. Shen et al. have elucidated gender differences in bladder cancer biology thought to be related to differential expression of sex steroid receptors on urothelial cells [29]. Specifically, the beta subunit of the estrogen receptor is the predominant receptor expressed in the majority of bladder cancers, and a positive correlation exists between degree of estrogen receptor expression and tumor grade and stage [29]. These gender differences in cancer biology may result in differences in cytologic morphology. Distinct patterns of chromosomal abnormalities between the genders have been described in other cancers and it is possible that the specific chromosomal aberrations detected by the UroVysion test result in improved sensitivity in men [30].

Proper stratification into relevant subgroups allows for recognition of important spectrum associations [31]. There is value in discerning between low grade and high grade lesions; high grade should be detected as early as possible, while the likelihood of missing such a tumor should be as low as possible. In high risk populations, sensitivity is more important than specificity because the consequences of a missed malignancy are great. FISH exhibits such properties in the smoking subgroup, whereas cytology does not have similar characteristics in the same population. Therefore, a clinician should give stronger consideration to FISH results than cytology results in smokers. Analogous spectrum effects can be seen for indication and cytology.

There are other patient populations were the risks of a procedure often outweigh the benefit. It is preferable for a urine test with a high specificity and low sensitivity in low grade disease to reduce the number of unnecessary invasive procedures. Age and cytology illustrate this effect because as the patient age increases, so does the specificity, with a reciprocal decrease in sensitivity. This should spare the elderly patient avoidable cystoscopies. The tradeoff would be that some tumors may be missed for a period of time, but the literature surrounding active surveillance suggest this is safe [32].

### Limitations

**Fig. 3:**
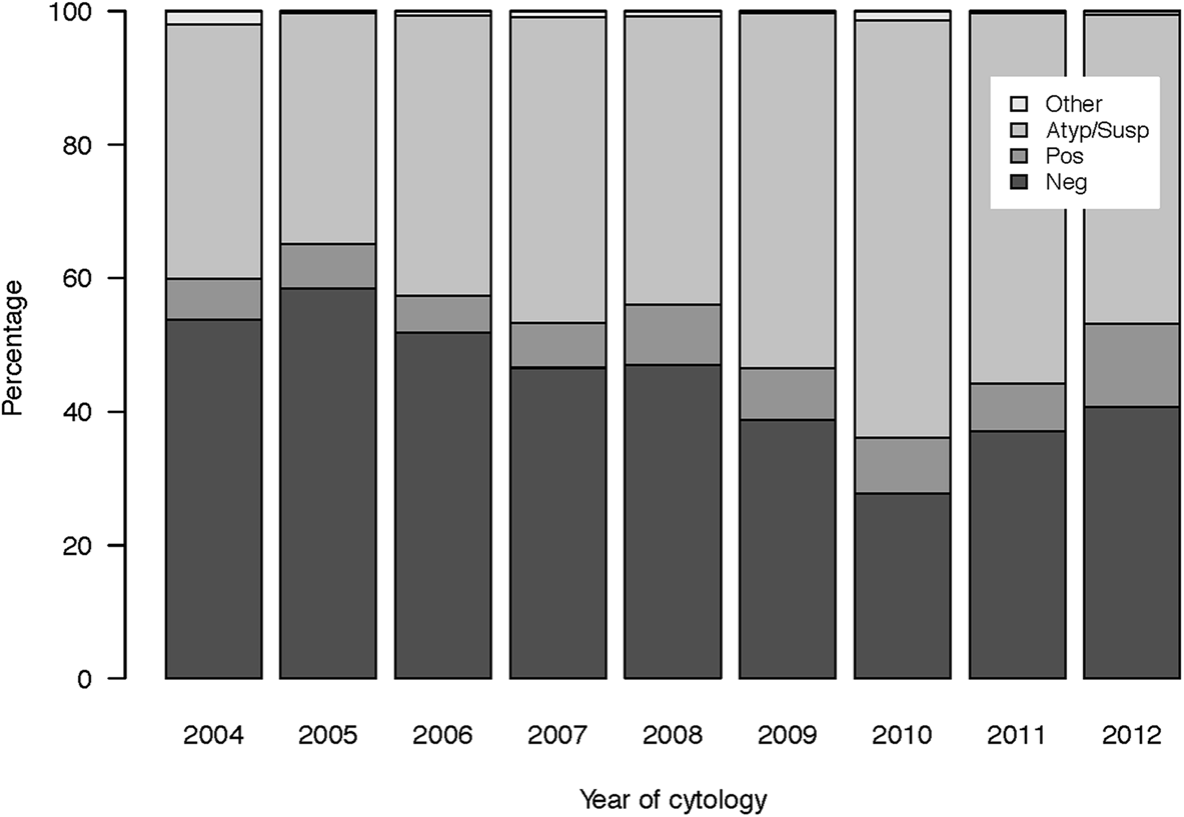
Classification of cytologies over time

Our study was retrospective, and longitudinal in nature leaving us unable to control for significant variables, such as the EORTC risk scores, that predict the probability of recurrence and progression of bladder cancer. With 19 % of cystoscopies in the cytology cohort classified as positive, this cohort was at higher risk for bladder cancer than the average US population. Additionally, while the sensitivity could have been improved with narrow band imaging or fluorescent cystoscopy, these technologies were not available at our institution for the entirety of the study period. For the purposes of our analyses, suspicious lesions on cystoscopy were classified as positive. When we correlated this classification with pathology, only 59 % of pathology specimens were found to have cancer, reflecting a limitation of this classification. However, when we performed a sensitivity analysis with suspicious cystoscopies classified as negative, our results were not significantly different, indicating a minimal impact of this limitation on the interpretation of the results. The data were collected over a 10 year time frame; so indications for using the tests have changed over time as have technique of verification of test results. Furthermore, more than one cytopathologist was involved over the period examined and literature suggests high inter-observer discrepancy, but this reflects the real world. Urine cytology has a low sensitivity and is highly operator-dependent in the setting of low grade disease [33]. In experienced hands, however, specificity is about 90 % [34]. Indeed, our own data supports this conclusion, and shows an increasing percentage of reported atypical/suspicious cytologies over time (Fig. 3).

## Conclusions

We are the first to show that urine-based bladder cancer tests display spectrum effects. The reporting of spectrum effects in diagnostic tests should become part of standard practice. Knowledge of these effects allows the physician to properly interpret the results and has a meaningful impact on a patient’s clinical care.

## Abbreviations

AJCC, American Joint Committee on Cancer; BC, Bladder cancer; CEP, Chromosome enumeration probes; DUMC, Duke University Medical Center; EORTC, European Organization for Research and Treatment of Cancer; FISH, Fluorescence in situ hybridization; GEE, Generalized estimating equations; GLMM, Generalized linear mixed models; IRB, Institutional Review Board; NMIBC, Non-muscle invasive bladder cancer; STARD, Standards for Reporting of Diagnostic Accuracy; UTUC, Upper tract urothelial carcinoma

## References

[1] Siegel RL, Miller KD, Jemal A (2015). Cancer statistics, 2015. CA Cancer J Clin.

[2] Raghavan D, Shipley WU, Garnick MB, Russell PJ, Richie JP (1990). Biology and management of bladder cancer. N Engl J Med.

[3] Network NCC (2015). NCCN Clinical Practice Guidelines in Oncology. Bladder Cancer. V.2.2015.

[4] Hall MC, Chang SS, Dalbagni G, Pruthi RS, Seigne JD, Skinner EC (2007). Guideline for the management of nonmuscle invasive bladder cancer (stages Ta, T1, and Tis): 2007 update. J Urol.

[5] Babjuk M, Burger M, Zigeuner R, Shariat SF, van Rhijn BW, Comperat E (2013). EAU guidelines on non-muscle-invasive urothelial carcinoma of the bladder: update 2013. Eur Urol.

[6] van der Aa MN, Steyerberg EW, Bangma C, van Rhijn BW, Zwarthoff EC, van der Kwast TH (2010). Cystoscopy revisited as the gold standard for detecting bladder cancer recurrence: diagnostic review bias in the randomized, prospective CEFUB trial. J Urol.

[7] Majewski T, Lee S, Jeong J, Yoon DS, Kram A, Kim MS (2008). Understanding the development of human bladder cancer by using a whole-organ genomic mapping strategy. Lab Invest.

[8] Swartz MA, Porter MP, Lin DW, Weiss NS (2006). Incidence of primary urethral carcinoma in the United States. Urology.

[9] Roupret M, Babjuk M, Comperat E, Zigeuner R, Sylvester RJ, Burger M (2015). European Association of Urology Guidelines on upper urinary tract urothelial cell carcinoma: 2015 update. Eur Urol.

[10] Ransohoff DF, Feinstein AR (1978). Problems of spectrum and bias in evaluating the efficacy of diagnostic tests. N Engl J Med.

[11] Elie C, Coste J (2008). A methodological framework to distinguish spectrum effects from spectrum biases and to assess diagnostic and screening test accuracy for patient populations: application to the Papanicolaou cervical cancer smear test. BMC Med Res Methodol.

[12] Mulherin SA, Miller WC (2002). Spectrum bias or spectrum effect? Subgroup variation in diagnostic test evaluation. Ann Intern Med.

[13] Bossuyt PM, Reitsma JB, Bruns DE, Gatsonis CA, Glasziou PP, Irwig LM (2003). Towards complete and accurate reporting of studies of diagnostic accuracy: the STARD initiative. Standards for Reporting of Diagnostic Accuracy. Clin Chem.

[14] Clark PE, Agarwal N, Biagioli MC, Eisenberger MA, Greenberg RE, Herr HW (2013). Bladder cancer. J Natl Compr Canc Netw.

[15] Kamat AM, Hegarty PK, Gee JR, Clark PE, Svatek RS, Hegarty N (2013). ICUD-EAU International Consultation on Bladder Cancer 2012: Screening, diagnosis, and molecular markers. Eur Urol.

[16] Karl A, Adejoro O, Saigal C, Konety B (2014). General adherence to guideline recommendations on initial diagnosis of bladder cancer in the United States and influencing factors. Clin Genitourin Cancer.

[17] Zhou XH (1998). Correcting for verification bias in studies of a diagnostic test’s accuracy. Stat Methods Med Res.

[18] Smith GD, Bentz JS (2010). “FISHing” to detect urinary and other cancers: validation of an imaging system to aid in interpretation. Cancer Cytopathol.

[19] Genders TS, Spronk S, Stijnen T, Steyerberg EW, Lesaffre E, Hunink MG (2012). Methods for calculating sensitivity and specificity of clustered data: a tutorial. Radiology.

[20] Fitzmaurice GM. Applied Longitudinal Analysis. Hobocken, New Jersey: Wiley; 2004.

[21] Hubbard AE, Ahern J, Fleischer NL, Van der Laan M, Lippman SA, Jewell N (2010). To GEE or not to GEE: comparing population average and mixed models for estimating the associations between neighborhood risk factors and health. Epidemiology.

[22] Taylor JA, Kuchel GA (2009). Bladder cancer in the elderly: clinical outcomes, basic mechanisms, and future research direction. Nat Clin Pract Urol.

[23] Chamssuddin AK, Saadat SH, Deiri K, Zarzar MY, Abdouche N, Deeb O (2013). Evaluation of grade and stage in patients with bladder cancer among smokers and non-smokers. Arab J Urol.

[24] Horstmann M, Todenhofer T, Hennenlotter J, Aufderklamm S, Mischinger J, Kuehs U (2013). Influence of age on false positive rates of urine-based tumor markers. World J Urol.

[25] Dimashkieh H, Wolff DJ, Smith TM, Houser PM, Nietert PJ, Yang J (2013). Evaluation of urovysion and cytology for bladder cancer detection: a study of 1835 paired urine samples with clinical and histologic correlation. Cancer Cytopathol.

[26] Fajkovic H, Halpern JA, Cha EK, Bahadori A, Chromecki TF, Karakiewicz PI (2011). Impact of gender on bladder cancer incidence, staging, and prognosis. World J Urol.

[27] Garg T, Pinheiro LC, Atoria CL, Donat SM, Weissman JS, Herr HW (2014). Gender disparities in hematuria evaluation and bladder cancer diagnosis: a population based analysis. J Urol.

[28] Straface E, Gambardella L, Brandani M, Malorni W. Sex differences at cellular level: "cells have a sex". Handb Exp Pharmacol. 2012;(214):49–65. 10.1007/978-3-642-30726-3_3 23027445

[29] Shen SS, Smith CL, Hsieh JT, Yu J, Kim IY, Jian W (2006). Expression of estrogen receptors-alpha and -beta in bladder cancer cell lines and human bladder tumor tissue. Cancer.

[30] Tabernero MD, Espinosa AB, Maillo A, Rebelo O, Vera JF, Sayagues JM (2007). Patient gender is associated with distinct patterns of chromosomal abnormalities and sex chromosome linked gene-expression profiles in meningiomas. Oncologist.

[31] Lachs MS, Nachamkin I, Edelstein PH, Goldman J, Feinstein AR, Schwartz JS (1992). Spectrum bias in the evaluation of diagnostic tests: lessons from the rapid dipstick test for urinary tract infection. Ann Intern Med.

[32] Tiu A, Jenkins LC, Soloway MS (2014). Active surveillance for low-risk bladder cancer. Urol Oncol.

[33] Sherman AB, Koss LG, Adams SE (1984). Interobserver and intraobserver differences in the diagnosis of urothelial cells. Comparison with classification by computer. Anal Quant Cytol.

[34] Raitanen MP, Aine R, Rintala E, Kallio J, Rajala P, Juusela H (2002). Differences between local and review urinary cytology in diagnosis of bladder cancer. An interobserver multicenter analysis. Eur Urol.

